# Extraction and Structural Analysis of Sweet Potato Pectin and Characterization of Its Gel

**DOI:** 10.3390/polym16141977

**Published:** 2024-07-10

**Authors:** Chunmeng Han, Xiangying Zhao, Liping Yang, Mingjing Yao, Jiaxiang Zhang, Qiangzhi He, Jianjun Liu, Liping Liu

**Affiliations:** 1 Shandong Food Ferment Industry Research & Design Institute, Qilu University of Technology (Shandong Academy of Sciences), Jinan 250013, China; hanchunmeng67@163.com (C.H.); xyzhao68@126.com (X.Z.); sdhyylp@163.com (L.Y.); mingjing1004@163.com (M.Y.); jxzh23@163.com (J.Z.); heqiangzhi@126.com (Q.H.); liujj-2000@163.com (J.L.); 2 School of Food Science and Engineering, Qilu University of Technology (Shandong Academy of Sciences), Jinan 250353, China

**Keywords:** sweet potato pomace, pectin extraction, structural analysis, gel properties

## Abstract

Pectin is widely used in the food and pharmaceutical industries. However, data on sweet potato pectin extraction and structural property analyses are lacking. Here, for the high-value utilization of agricultural processing waste, sweet potato residue, a byproduct of sweet potato starch processing, was used as raw material. Ammonium oxalate, trisodium citrate, disodium hydrogen phosphate, hydrochloric acid and citric acid were used as extractants for the pectin constituents, among which ammonium oxalate had a high extraction rate of sweet potato pectin, low ash content and high molecular weight. Structural and gelation analyses were conducted on ammonium oxalate-extracted purified sweet potato pectin (AMOP). Analyses showed that AMOP is a rhamnogalacturonan-I-type pectin, with a molecular weight of 192.5 kg/mol. Chemical titration and infrared spectroscopy analysis confirmed that AMOP is a low-ester pectin, and scanning electron and atomic force microscopy demonstrated its linear molecular structure. Gelation studies have revealed that Ca^2+^ is the key factor for gel formation, and that sucrose significantly enhanced gel hardness. The highest AMOP gel hardness was observed at pH 4, with a Ca^2+^ concentration of 30 mg/g, pectin concentration of 2%, and sucrose concentration of 40%, reaching 128.87 g. These results provide a foundation for sweet potato pectin production and applications.

## 1. Introduction

Pectin acts as a hydrating agent and adhesive in plant cells, helping to maintain cell shape and provide structural support. Moreover, it is a naturally occurring, acidic heteropolysaccharide with a branched structure. Based on the structure of the main chain and composition of the side chains, pectin molecules typically have four structural types. Homogalacturonan (HG) consists of a main chain composed of repeating units of galacturonic acid (Gal-A) without side chains. Rhamnogalacturonan-I (RG-I) consists of repeating units of Gal-A and rhamnose (Rha) disaccharides in the main chain. At the C-4 position of Rha, either β-1,4-D-galactose or α-1,5-L-arabinose forms a single side chain [1]. Rhamnogalacturonan-II (RG-II) shares the same main-chain structure as HG and is composed of repeating units of Gal-A. However, nonhomogeneous side chains consisting of various monosaccharides, such as Rha, galactose (Gal), glucuronic acid (Glc-A), methylated Glc-A, and other rare carbohydrates, are attached to C-2 and C-3 of the backbone [2]. Xylose Gal-A was first discovered in pollen and is primarily present in plant reproductive organs. Its main chain consists of Gal-A with xylose (Xyl) substituted at O-3 or O-4 of the Gal-A residue. Further, it is a “substituted galacturonic acid” form. Pectin structural type proportions are related to the plant from which it originates. Generally, the main structural type is HG, with the RG-I structural type accounting for approximately 20–35% and the RG-II type accounting for approximately 10% [3]. Furthermore, based on the degree of methylation of galacturonic acid residues in pectin molecules, pectin can be classified into low-ester pectin and high-ester pectin. Low-ester pectin has only a small number of galacturonic acid residues that are methylated. High-ester pectin, on the other hand, has most or all of the galacturonic acid residues methylated [4].

From a functional point of view, pectin is a substance that forms gels and is widely used for food and pharmaceutical applications. In the food industry, it is used to process low-calorie, low-sugar functional health foods [5]. In the biomedical field, pectin can be used for the targeted delivery of bioactive substances, promotion of wound healing, and regulation of the intestinal microbiota composition [6]. In addition, in recent years, many physiological functional properties of pectin have been elucidated, such as its antioxidant, cholesterol-reducing, and tumor growth- and metastasis-inhibiting effects [7]. Commercial pectin is mainly produced from the byproducts of citrus peels and apple pomace. However, the extraction of pectin from industrial waste byproducts, such as sugar beet pulp and potato pulp, also represents a potential source of this compound [8]. The main methods used to extract pectin from plant materials include water, acid, and salt extraction [9]. Moreover, the chemical properties of extracted pectin depend on the extraction conditions and source materials. For example, the use of an acid extraction method to extract pectin can result in the partial hydrolysis of neutral sugars on the pectin side chains and a decrease in its molecular weight [10]. As such, corresponding changes in the apparent properties of pectin, such as its viscosity and gel characteristics, also occur [11].

Sweet potatoes are an important staple crop in China. According to statistics from the Food and Agriculture Organization of the United Nations, total sweet potato production in China reached 51.264 million tons in 2020, making it the world’s largest producer of sweet potatoes [12]. In addition to direct consumption, starch processing is one of the primary processing methods used for sweet potatoes. Sweet potato pomace is a byproduct of this process, and currently, it is often accumulated in large quantities or sold as cheap feed without being effectively utilized to enhance its value. Sweet potato pomace is rich in pectin, with a content of approximately 20% (on a dry basis), making it a potential alternative raw material for producing commercial pectin [13].

Sweet potato residues are abundant, inexpensive, and rich in pectin. However, there are relatively few reports of studies on the extraction of pectin from sweet potato residues, particularly regarding its structural properties. For example, Zhang et al. [14] studied the extraction of pectin from sweet potato pomace using disodium hydrogen phosphate as an extractant, achieving a yield of 10.24% and a degree of esterification of 11.2%. This team also optimized the sweet potato pectin extraction process using citric acid as the extractant, resulting in a pectin extraction rate of only 5.09% and a degree of esterification of 29.45% [15]. In this study, sweet potato residues, a byproduct of sweet potato starch processing, were used as raw materials, and the effects of five extractants, namely ammonium oxalate, trisodium citrate, disodium hydrogen phosphate, hydrochloric acid, and citric acid, on the extraction rate and basic components of sweet potato pectin were investigated. The structure and gel properties of sweet potato pectin extracted and refined using ammonium oxalate were also studied. This study provides a theoretical basis for the production and application of sweet potato pectin, promoting the comprehensive utilization of sweet potato resources.

## 2. Materials and Methods

### 2.1. Materials and Chemicals

Sweet potato pomace (a byproduct of sweet potato starch processing) was provided by Shandong Sishui Lifeng Food Co., Ltd. (Jining, China). Gal, Rha, glucose (Glc), mannose (Man), fucose (Fuc), Xyl, arabinose (Ara), Gal-A, and Glc-A standards were purchased from Sigma (St. Louis, MO, USA). Orange peel pectin was purchased from Sigma. Trifluoroacetic acid was purchased from Shanghai Aladdin Bio-Chem Technology Co., Ltd. (Shanghai, China). All other reagents and solvents were purchased from Sinopharm Chemical Reagent Co., Ltd. (Shanghai, China).

### 2.2. Pre-Treatment of Sweet Potato Pomace

An amount of 500 g of sweet potato residue was placed in a filter bag with a 200-mesh filter. The residue was continuously washed with running water until the soluble solids content in the filtrate reached 0%, and then it was squeezed dry. The dry-squeezed sweet potato residue was mixed with water at a solid-to-liquid ratio of 1:20 (g/mL) and stirred thoroughly. Subsequently, 0.09 mL of high-temperature amylase (150,000 IU/mL) was added, and the mixture was liquefied at 90 °C for 20 min. After cooling to 60 °C, 0.09 mL of glucoamylase (60,000 IU/mL) was added, and the mixture was saccharified for 4 h. The liquefied and saccharified sweet potato residue was then washed and filtered multiple times until the soluble solid content in the filtrate reached 0%, and was squeezed dry to obtain starch-free sweet potato residue. The resulting residue was frozen for future use.

### 2.3. Extraction of Sweet Potato Pectin

The extraction of pectin was conducted as follows: When using a salt extractant, 100 g of de-starched wet sweet potato residue was weighed, and 263 mL of water was added at a solid-to-liquid ratio of 1:20 (g/mL) on a dry basis. Trisodium citrate, ammonium oxalate, and disodium hydrogen phosphate were added to the system as extractants at concentrations of 0.5% (*w*/*w*), 0.8% (*w*/*w*), and 1.0% (*w*/*w*) of the total mass of the system, respectively. The mixture was thoroughly stirred and maintained at 90 °C for 2 h. For acid extraction, 100 g of de-starched wet sweet potato residue was weighed, and 263 mL of water was added at a solid-to-liquid ratio of 1:20 (g/mL) on a dry basis. Subsequently, 3 mL of 1:1 hydrochloric acid and 14 g of citric acid were added to adjust the system pH to 2. The mixture was then stirred and maintained at 80 °C for 1 h.

Following the salt extraction and acid extraction processes, the sweet potato residue was continuously washed with water. Solid–liquid separation was performed via vacuum filtration until the soluble solids content in the filtrate reached 0%. The filtrates were then combined. The combined filtrate was dialyzed using dialysis bags with a molecular weight cutoff of 5 kDa until the conductivity reached 2.9 μs/cm. The dialyzed filtrate was subsequently concentrated to one-third of its original volume using rotary evaporation. Under constant stirring, the concentrated solution was thoroughly mixed with a two-fold volume of 95% anhydrous ethanol, chilled overnight at 4 °C, and centrifuged at 4200× *g* rpm for 15 min. The precipitate was collected and dried under freeze-drying conditions for weighing. The pectin yield was calculated using Formula (1):

(1) $$Pectin\;yield\;\left(\%\right)=\frac{Weight\;of\;dried\;extracted\;pectin}{Dry\;basis\;weight\;of\;sweet\;potato\;residue}\times 100$$

### 2.4. Refinement of Sweet Potato Pectin

Lyophilized ammonium oxalate-extracted sweet potato pectin was dissolved in an appropriate amount of water, mixed homogeneously with glucoamylase (20 IU/g pectin), and then incubated at 60 °C for 6 h. The pectin solution was dialyzed repeatedly using dialysis bags with a molecular weight cutoff of 5 kDa until the glucose content in the pectin solution reached 0 mg/dL (determined using a biosensor analyzer). The dialysate was freeze-dried to obtain ammonium oxalate extracted and refined sweet potato pectin (AMOP), which was then used for structural characterization and gel properties.

### 2.5. Basic Composition

The moisture and ash contents of the pectin were determined according to the methods outlined in GB 5009.3-2016 and GB 5009.4-2016 [16,17], respectively. The protein content was determined using an automatic Kjeldahl nitrogen analyzer [18]. The glucose content was measured using a biosensor analyzer.

### 2.6. Monosaccharide Composition

Monosaccharide components were analyzed using an LC-20A liquid chromatograph (Shimadzu, Tokyo, Japan) equipped with a UV detector (SPD-20A) and a C18 column (4.6 × 250 mm, 5 μm). Pectin (0.05 g) was hydrolyzed in 4 M trifluoroacetic acid at 110 °C for 2 h. Following hydrolysis, the pH was adjusted to 7 using sodium hydroxide, and the volume was adjusted to 20 mL with water. The resulting hydrolysate (1 mL) was mixed with 1 mL of 0.3 mol/L sodium hydroxide and 1 mL of a 0.5 mol/L 1-phenyl-3-methyl-5-pyrazolone methanol solution and reacted at 70 °C for 70 min. After cooling, 1 mL of 0.3 mol/L acetic acid was added, and the volume was adjusted to 10 mL. Subsequently, 2 mL of the derivatization solution was extracted with chloroform. The aqueous layer was retained, and the sample was filtered through a 0.22 μm filter for detection. The mobile phase consisted of 50 mmol/L potassium dihydrogen phosphate A and acetonitrile B, with a flow rate of 1.0 mL/min and a column temperature maintained at 40 °C. The gradient program was as follows: 85% A and 15% B at 2 min, 83% A and 17% B at 8 min, 82% A and 18% B at 20 min, 60% A and 40% B at 36 min, 60% A and 40% B at 40 min, with a return to 85% A and 15% B at 42 min, concluding at 47 min.

### 2.7. Molecular Weight

The molecular weight of pectin was determined using an Agilent 1260 Infinity II GPC system (Agilent, Santa Clara, CA, USA). The system was equipped with a chromatographic column (PL aquagel-OH MIXED-H 8 μm), a differential refractive index detector, and a multi-angle laser light scattering detector. Polyethylene oxide (PEO) served as a calibration standard for method validation, possessing a molecular weight of 1000 kDa. Pectin (10 mg) was carefully weighed, dissolved in ultrapure water, and thoroughly stirred until fully dissolved. The resulting solution was adjusted to a volume of 10 mL and filtered through a 0.22 μm filter. During analysis, 0.1 M sodium nitrate was employed as the eluent, operating at a flow rate of 1.0 mL/min, while maintaining the column temperature at 30 °C.

### 2.8. Degree of Esterification (DE) and Methoxy Content (DM)

The DE for pectin was determined according to the method described by Kazemi et al. [19], with some modifications. The pectin sample (0.1 g) was initially mixed with 1 mL of ethanol and subsequently added to 100 mL of distilled water, followed by stirring at room temperature for 2 h. Two drops of phenolphthalein indicator were introduced, and the resulting solution was titrated with a 0.1 mol/L sodium hydroxide standard solution until a pink color appeared, marking the endpoint. The volume of sodium hydroxide solution consumed at this point (V_1_, mL) was recorded. Following the titration, 10 mL of a 0.1 mol/L sodium hydroxide standard solution was added to the solution, which was stirred at room temperature for 15 min. Subsequently, 10 mL of a 0.1 mol/L standard hydrochloric acid solution was introduced, and a titration was conducted again using the 0.1 mol/L sodium hydroxide standard solution to determine the volume consumed at the endpoint (V_2_, mL). DE was calculated using Formula (2). The methoxy content in pectin molecules reflects the degree of methylation of galacturonic acid units, also known as the degree of esterification. The maximum theoretical value of methoxy groups in pectin is 16.3%. If this value represents 100% esterification, the conversion relationship between DM and DE is calculated using Equation (3).

(2) $$DE\;(\%)=\frac{V_{2}}{\left(V_{2}-V_{1}\right)}\times 100$$

(3) DM (%)=DE×0.163

### 2.9. Fourier-Transform Infrared Spectroscopy (FTIR)

The dried pectin sample was mixed and ground with pre-dried KBr at a ratio of 1:100, pressed into pellets, and analyzed using a VERTEX 70 Fourier-transform infrared spectrometer (Bruker, Rheinstetten, Germany) at 22 °C, with air as the background and full-range scanning. Spectra were collected through scanning, 64 times, with a spectral resolution of 4 cm^−1^, covering the range of 4000–500 cm^−1^. The same procedure was used for the orange peel pectin standard.

### 2.10. Scanning Electron Microscopy (SEM)

Pectin samples were fixed on a sample stage and transferred to an S-4800 scanning electron microscope (Hitachi, Tokyo, Japan) for observation after being sprayed with gold under surface vacuum conditions.

### 2.11. Atomic Force Microscopy (AFM)

A pectin solution with a concentration of 0.5 µg/mL was prepared, and 5 μL of this was gently dropped onto a mica slide. Then, it was gently rotated to ensure uniform dispersion of the solution. The mica slides were placed in a dry, room-temperature environment. Samples were observed using a Multimode 8 atomic force microscope (Bruker, Rheinstetten, Germany).

### 2.12. Gelling Properties

#### 2.12.1. Formation Factors of Sweet Potato Pectin Gel

Pectin (1 g) was dissolved in deionized water under stirring to prepare pectin solutions with concentrations of 0.2%, 0.5%, 1.0%, 1.5%, 2.0%, and 2.5% (*w*/*w*), which were subsequently transferred into beakers. These solutions were then stored at 4 °C for 24 h to observe the occurrence of gel formation solely attributable to varying concentrations. Following this, solutions containing pectin concentrations of 0.2%, 0.5%, 1.0%, 1.5%, 2.0%, and 2.5% (*w*/*w*) were heated to 80 °C, and 60% sucrose was added under continuous stirring until complete dissolution. The solutions were then stored at 4 °C for 24 h to observe the gel formation induced by the addition of high-concentration sugar. Additionally, at 80 °C, 40 mg/g Ca^2+^ was introduced into pectin solutions with concentrations of 0.2%, 0.5%, 1.0%, 1.5%, 2.0%, and 2.5% (*w*/*w*), with continuous stirring until fully dissolved. These solutions were also stored at 4 °C for 24 h to observe the gel formation that was solely the result of the addition of Ca^2+^.

#### 2.12.2. Texture Performance

The gel properties of sweet potato pectin were investigated by selecting different pectin solution concentrations, Ca^2+^ concentrations, pH levels, and sucrose contents based on factors affecting pectin gel formation. Pectin solutions of varying concentrations (0.2%, 0.5%, 1%, 1.5%, 2.0%, 2.5%, *w*/*w*) were prepared, with the addition of a 40 mg/g CaCl_2_ solution under conditions of pH 4.0 and 80 °C. At a concentration of 2.0%, pH 4, and 80° C, different Ca^2+^ concentrations (10 mg/g, 20 mg/g, 30 mg/g, 40 mg/g, 50 mg/g) were introduced. The pH levels were adjusted from 2 to 7 under conditions of 2.0% pectin concentration, 30 mg/g Ca^2+^, and 80 °C. Different concentrations of sucrose (10%, 20%, 30%, 40%, 50%, *w*/*w*) were added under conditions of 2.0% pectin concentration, 30 mg/g Ca^2+^, 80 °C, and pH 4. The gels prepared under these various conditions were stored at 4 °C for 24 h. The gel texture of the prepared gel samples was measured using a CT-3 texture analyzer (Brookfield, WI, USA). Test parameters were as follows: compression mode, with a TA 10 probe, compression speed of 1.0 mm/s, test speed of 1.0 mm/s, post-contact speed of 1.0 mm/s, and measurement height of 4 mm.

### 2.13. Statistical Analysis

Each experiment was repeated at least three times. Statistical analysis of the experimental data was conducted using SPSS 21.0, and the results are presented as the mean ± standard deviation.

## 3. Results and Discussion

### 3.1. Effects of Extractants on the Extraction of Sweet Potato Pectin

Currently, the methods used for extracting pectin from plant materials mainly include acid, salt, and water extraction. However, different extraction methods significantly affect the extraction yield and properties of pectin. Acids can hydrolyze complex cross-linking networks in the cell wall, facilitating the dissolution and release of pectin. Salts can effectively chelate metal ions such as Ca^2+^ in the cell wall, thereby disrupting the connections between pectin molecules and metal ions, promoting the release of pectin. Ammonium oxalate, disodium hydrogen phosphate, trisodium citrate, hydrochloric acid, and citric acid were selected as extractants in order to compare their effects on the extraction rate and basic components of sweet potato pectin.

The extraction rates and basic components of sweet potato pectin extracted using the different extractants are listed in Table 1. The maximum extraction rate of salt extracted pectin was 14.42% and that of acid extracted pectin was about 8.91%. The rate of pectin extracted using salt extractants was much higher than that using acid extractants. The highest molecular weight of pectin extracted using the three salt extractants was 182.5 kg/mol, whereas that using the two acid extractants was 152.5 kg/mol. This phenomenon arises because salt extractants can effectively extract pectin from plant materials under mild conditions without causing significant pectin degradation. However, pectin chains degrade in acidic environments, especially the degradation of neutral sugar side chains, leading to a decrease in pectin molecular weight [20]. Among the three salt extractants, ammonium oxalate, at the same concentration as the salt extractant, resulted in the highest extraction rate, lowest ash content, and largest molecular weight. Additionally, at pH 2, the extraction rate of pectin extracted using hydrochloric acid was 8.91%, higher than that when using citric acid, which was 7.61%. However, to achieve the same pH as hydrochloric acid, approximately 5% citric acid needed to be added to the extraction solution. This not only increases the cost of the extractant, but also increases pollutant emissions. Therefore, citric acid was deemed unsuitable for commercial pectin extraction.

For a more accurate analysis of the structural properties of sweet potato pectin, pectin extracted with ammonium oxalate was enzymatically hydrolyzed and dialyzed to obtain refined ammonium oxalate-extracted purified sweet potato pectin (AMOP). The basic compositions of the refined AMOP are listed in Table 2. After refinement, the ash and protein content of the pectin decreased, whereas the molecular weight increased, making it more suitable for studying the structural properties of pectin.

### 3.2. Monosaccharide Composition Analysis of AMOP

The monosaccharide composition of AMOP is shown in Table 3. The Gal-A, Ara, Gal, and Rha contents in AMOP were 41%, 21.08%, 29.84%, and 6.12%, respectively. In addition, it was found to contain small amounts of Glc and Fuc. Gal-A and Rha are derived from the backbone of HG and RG-I, while Gal and Ara comprise the neutral sugar side chains linked to the backbone of RG-I. The content of Gal-A is below 50%, which may indicate a relatively low proportion of HG in the AMOP; furthermore, the content of Gal is significantly higher than that of Ara, suggesting that the RG-I region may be highly branched with galactan or arabinogalactan.

To further elucidate the structure of AMOP, the following proportions have been noted in the literature: RG-I (%) = 2 Rha + Ara + Gal and HG (%) = GalA–Rha [21]. According to this relationship, the proportions of HG and RG-I structures in AMOP could be calculated as 34.88% and 63.16%, respectively, indicating that AMOP is an RG-I-type pectin. In addition, the ratio of Rha to Gal-A can indirectly indicate the relative contents of RG-I and HG in pectin. For pectin polysaccharides, mainly composed of RG-I structures, the Rha/Gal-A ratio typically falls within the range of 0.05–1. The Rha/Gal-A ratio of AMOP was 0.14, further indicating that RG-I structures predominated in AMOP. In the RG-I domain, Rha residues are typically linked to Gal and Ara side chains. The molar ratio of (Gal + Ara) to Rha can be used to evaluate the degree of branching in the RG-I domain [22]. The molar ratio of AMOP was 8.32%, indicating that it was rich in Ara and Gal side chains, and that the presence of these side chains enriches the structure and function of pectin.

### 3.3. Molecular Weight Analysis of AMOP

The molecular weight of pectin is another important parameter for its structural analysis. The molecular weights of sweet potato pectin are listed in Table 4. The weight-average molecular weight (Mw) of AMOP was 192.5 kg/mol, the number-average molecular weight (Mn) was 174.7 kg/mol, and the polydispersity index (PD) was 1.10. The PD value reflects the uniformity of the molecular weight distribution of the polymer, the smaller the PD value, the more uniform the molecular weight distribution. The PD of AMOP was close to 1, indicating a narrow molecular weight distribution and relatively uniform molecular size, suggesting high pectin purity. The molecular weight of AMOP was lower than that reported by Mu et al. [23], who used sodium hexametaphosphate for extraction (6.45 × 10^5^ g/mol); this could be related to the sweet potato variety and the extraction and purification methods used.

### 3.4. DE and DM Analysis of AMOP

In its natural state, some Gal-A carboxyl groups in the main chain of pectin are esterified with methanol or acetyl groups. The degree of Gal-A esterification in pectin molecules is related to its plant source, the growth cycle of the plant, and the extraction process [24]. Additionally, the DE and DM of pectin are important factors affecting its physicochemical properties. The DE and DM of AMOP are shown in Table 4. The DE of AMOP, measured via titration was 11.76%, and the methoxy content was 1.92%. Sweet potato pectin is similar to sunflower head pectin and potato pectin, belonging to a class of naturally low-ester pectins [25,26].

### 3.5. FTIR Analysis of AMOP

**Figure 1:**
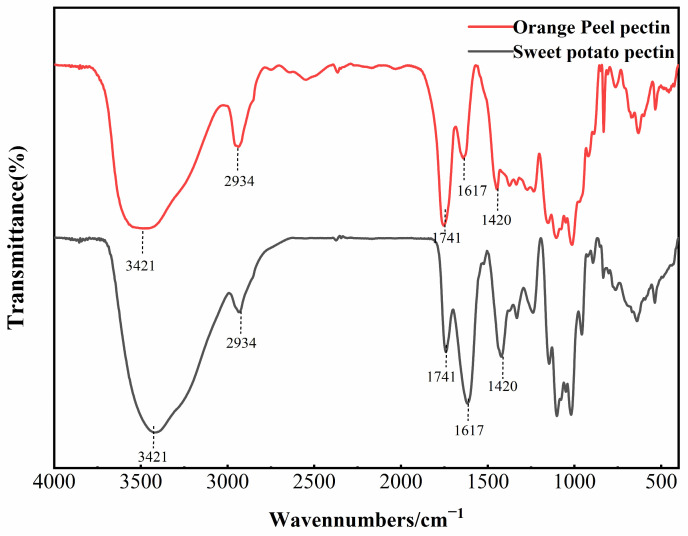
Fourier-transform infrared spectroscopy (FTIR) spectrum of ammonium oxalate-exracted purified sweet potato pectin (AMOP).

Infrared spectroscopy is typically used to identify the main functional groups in pectin structures. The FTIR spectrum of AMOP is shown in Figure 1, and it can be observed that its major absorption peaks were similar to those of citrus pectin. The strong and broad peak at 3421 cm^−1^ is caused by O–H stretching vibrations. The peak at around 2934 cm^−1^ corresponds to the absorption of the stretching and the stretching vibrations of C–H groups (CH, CH_2_ and CH_3_) in pectin [27].The peak at 1741 cm^−1^ corresponds to the stretching vibration of the carbonyl group in the methyl-esterified carboxyl groups [28]. Meanwhile, the peaks at 1617 cm^−1^ and 1420 cm^−1^ are attributed to the asymmetric and symmetric stretching vibrations of free carboxyl groups, respectively, confirming the presence of uronic acids [29]. The region from 800 to 1300 cm^−1^ is known as the “fingerprint region” of polysaccharide FTIR spectra, where the absorption peaks are dense and highly overlapping, reflecting the types and linkage modes of sugar residues [30]. Peaks in the range of 1000–1200 cm^−1^ correspond to the skeletal vibrations of C–O and C–C bonds in glycosidic linkages and pyranose rings, which are characteristics of polysaccharides [31]. The ratio of the peak area at 1741 cm^−1^ to the sum of the peak areas at 1617 cm^−1^ and 1741 cm^−1^ can reflect the degree of pectin esterification [32]. In commercial citrus pectin, the peak area at 1741 cm^−1^ is greater than that at 1617 cm^−1^, which is characteristic of highly esterified pectin. However, in the case of AMOP, the peak area at 1741 cm^−1^ was significantly lower than that at 1617 cm^−1^. The DE of AMOP calculated from the peak areas was 13.79%, which is close to the value determined via titration, further confirming that AMOP is a low-ester pectin.

### 3.6. Microscopic Morphology Analysis of AMOP

The microscopic morphology of pectin is a fundamental characteristic. SEM and AFM were used to observe the microscopic morphology of the freeze-dried samples and their molecular morphologies in an aqueous solution.

#### 3.6.1. SEM Observation of AMOP

**Figure 2:**
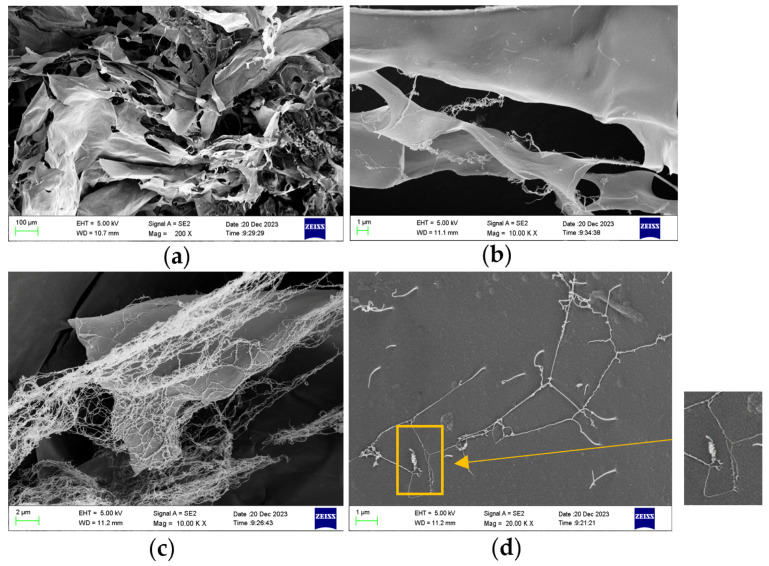
Scanning electron microscopy (SEM) image of ammonium oxalate-extracted purified sweet potato pectin (AMOP). (**a**) Magnification 200, (**b**) magnification 10,000, (**c**) magnification 10,000, and (**d**) magnification 20,000.

The AMOP sample was dissolved in water and freeze-dried for the SEM observation of its microstructure. An SEM image of the AMOP is shown in Figure 2, revealing a lamellar structure with a smooth surface. Additionally, a network-like structure attached to the surface of the lamellae was observed, which could have resulted from the low concentration of the pectin solution used during freeze-drying. Figure 2d shows the observation of some helical and fibrous aggregates, which can be regarded as macroscopic representations of molecular structure. These can reflect the linear morphology features of AMOP from the side.

#### 3.6.2. AFM Observation of AMOP

**Figure 3:**
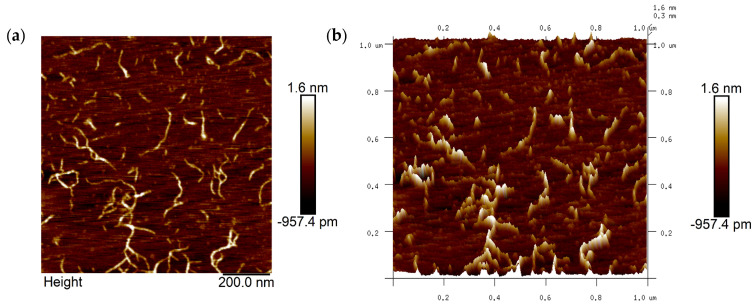
Atomic force microscopy (AFM) image of ammonium oxalate-extracted purified sweet potato pectin (AMOP). (**a**) Two-dimensional AFM analysis and (**b**) three-dimensional AFM analysis.

AFM can be used to collect microtopographic information about the surfaces of objects and is an important tool for studying the conformation of pectin polysaccharide molecules. The image of the sample obtained under AFM after dropping 0.5 µg/mL of AMOP solution onto a mica sheet is shown in Figure 3. The image shows pectin molecules in a fibrous form in a dilute solution, and some branching-like structures can be observed. From the analysis of their length and height, these fibers were considered aggregates or supramolecular assemblies of multiple pectin molecules, formed either via main chain branching or molecular aggregation, as the length of the neutral sugar side chains relative to the pectin molecules can be neglected.

### 3.7. Gelling Property Analysis of AMOP

**Figure 4:**
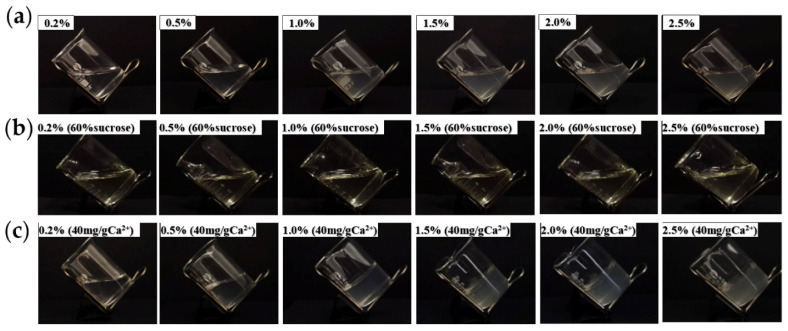
Effects of the ammonium oxalate-extracted purified sweet potato pectin (AMOP) concentration, Ca^2^⁺ addition, and sucrose addition on the gelation of sweet potato pectin. (**a**) Gel formation diagram at 0.2–0.5% pectin concentration, (**b**) 0.2–0.5% pectin concentration with sucrose gel formation, and (**c**) gel formation with the addition of Ca^2+^ at 0.2–0.5% pectin concentration.

The ability of pectin to form a gel is an important characteristic, and its gel properties are crucial for its applications in the food and pharmaceutical industries. In addition to being influenced by its structure, the gel properties of pectin are affected by environmental factors, such as ion concentration, pH, and sucrose concentration [33]. The effects of the pectin concentration, Ca^2+^ addition, and sucrose addition on AMOP gel formation are shown in Figure 4. At pH 4, when the pectin concentration ranged from 0.2% to 2.5%, the solution in the beaker was in a tilted state, indicating that increasing the pectin concentration alone cannot result in gel formation. Additionally, 60% sucrose addition under these conditions did not induce gel formation. However, the addition of 40 mg/g Ca^2+^ alone caused gel formation with 1–2.5% AMOP solutions. This gel phenomenon is consistent with the structural properties of AMOP as a low-ester pectin.

#### 3.7.1. Effect of Pectin Concentration on the Texture of AMOP Gels

Under the conditions of a Ca^2^⁺ concentration of 40 mg/g and pH of 4, the influence of the AMOP concentration on the gel texture was investigated. The effect of the AMOP concentration on the gel texture of sweet potato pectin is shown in Table 5. When the pectin concentration was 0.2%, a gel structure could not be formed owing to the incomplete exchange between Ca^2^⁺ and pectin molecules. When the pectin concentration was increased to 0.5%, the gel hardness was 4.6 g, and a stable cured gel could not be formed. With an increase in the pectin concentration from 0.5% to 2.0%, the gel hardness, viscosity, elasticity, cohesiveness, and chewiness all tended to increase. When the pectin concentration reached 2%, the gel hardness reached a maximum value of 102.20 g. This is because, under the premise of a fixed Ca^2^⁺ concentration, as the pectin concentration increases, the number of carboxyl groups available for exchange in the solution increases accordingly, strengthening hydrogen bonding and hydrophobic interactions and thereby enhancing the strength and gel formation rate [34]. However, when the pectin concentration reached 2.5%, the gel hardness decreased to 99.53 g. This phenomenon is attributed to an excessively high concentration, which could lead to overly tight molecular interactions, triggering repulsion effects and adversely affecting the stability and strength of the gel.

#### 3.7.2. Effect of Ca^2+^ Concentration on the Texture of AMOP Gels

Ca^2^⁺ plays a crucial regulatory role in the formation of low esterified pectin gels. By controlling the concentration of Ca^2^⁺, the strength and other textural properties of pectin gels can be effectively adjusted. Under the conditions of a 2% AMOP solution and a pH of 4, the effect of the Ca^2^⁺ concentration on the gel texture of AMOP was studied. The effect of the Ca^2^⁺ concentration on the gel texture of AMOP is shown in Table 6. When no Ca^2^⁺ was added to the pectin solution, a stable gel could not be formed. When the Ca^2^⁺ concentration was 10 mg/g, the pectin gel hardness was 29.67 g. Moreover, as the Ca^2^⁺ concentration was increased to 30 mg/g, the gel hardness increased to 105.39 g, showing a significant upward trend. Within the range of 10 mg/g to 30 mg/g, Ca^2^⁺ could effectively bind the Gal-A residues on the pectin molecules, forming an “egg-box” model connection, thereby promoting gel formation [35]. At the same time, with the increase in the Ca^2^⁺ concentration, hydrophilic groups increasingly bind to Ca^2^⁺, thus exposing more hydrophobic groups, enhancing the hydrophobic interactions between pectin molecules, and further improving the gel strength. When the Ca^2^⁺ concentration was in the range of 40–50 mg/g, the pectin gel hardness tended to balance, with values of 102.20 and 103.37 g, respectively. Within this concentration range, the binding of Ca^2^⁺ to pectin molecules reached its saturation point, and, thus, the gel hardness no longer varied substantially with increasing Ca^2^⁺ concentration.

#### 3.7.3. Effect of pH on the Texture of AMOP Gels

pH is one of the main factors affecting the gel formation of low-ester pectin, as it directly controls the degree of carboxyl group dissociation in pectin molecules. The effect of pH on the gel structure of AMOP, when changing the pH under conditions of 2% AMOP and 30 mg/g Ca^2^⁺ concentrations, is shown in Table 7. At pH 2, the gel hardness was 25.20 g and at pH 3 it was 39.20 g. Although gel formation was observed, the gel hardness was low. This is because, under overly acidic conditions, the carboxyl groups in pectin mainly exist as non-ionized carboxyl groups, which hinders their ability to effectively form stable “eggshell” model associations with Ca^2^⁺. Within the pH range of 3.5–5, the gel texture characteristics of AMOP differed significantly from those formed under other pH conditions, with the gel hardness reaching its maximum value of 105.39 g at pH 4. Between pH 3.5 and 5, the number of uncharged carboxyl groups in the pectin molecules gradually decreased, whereas the number of negatively charged carboxyl groups increased. This enhanced the interaction between pectin molecules and Ca^2^⁺, thereby promoting the formation of a more stable gel. When the pH was increased to 6–7, the gel hardness decreased sharply compared with that at pH 3.5–5. At pH 6, the gel hardness was 88.20 g, and at pH 7, it was 85.71 g. This decrease in gel hardness was due to the gradual reduction in the concentration of H⁺ ions at higher pH values, resulting in a decrease in the number of hydrogen bonds. This weakened the binding capacity of the free carboxyl groups. Excessive carboxyl groups can increase the repulsive forces between molecules, leading to decreased gel stability and deteriorated gel performance.

#### 3.7.4. Effect of Sucrose Concentration on the Texture of AMOP Gels

Highly esterified pectin molecules contain fewer free carboxyl groups. To reduce water activity and promote the formation of hydrogen bonds between pectin molecules, it is necessary to add over 55% (*w*/*v*) sucrose to form a gel. AMOPs are low-ester pectins. The addition of high concentrations of sucrose alone did not yield a stable gel structure. However, Ca^2^⁺ addition led to the formation of a stable gel. The effect of the sucrose concentration on the gel texture of AMOP under the conditions of pH 4, a pectin concentration of 2%, and a Ca^2^⁺ concentration of 30 mg/g, is shown in Table 8. While increasing sucrose concentration in the pectin solution from 0% to 40%, the gel hardness increased from 105.39 to 128.87 g. The addition of sucrose reduces the degree of solvation of pectin molecular chains, facilitating interactions between the pectin molecular chains, and thereby promoting the formation of hydrogen bonds and gels [36]. However, when the sucrose concentration in the pectin solution reached 50%, the gel hardness decreased to 120.00 g. At high sucrose concentrations, sucrose molecules hinder the interaction between pectin molecules, reducing the hydrogen bonds between water and pectin molecules, thereby affecting the formation of molecular networks and resulting in a decrease in the gel strength.

## 4. Conclusions

Five different extractants (ammonium oxalate, trisodium citrate, disodium hydrogen phosphate, hydrochloric acid, and citric acid) were compared to determine their effects on the extraction rate and basic composition of sweet potato pectin. The results show that salt extraction resulted in higher extraction yields and greater molecular weight of extracted pectin than acid extraction. The structural properties of the AMOP were then analyzed. AMOP was determined to be an RG-I-type pectin, accounting for 63.16% of its structure. The molecular weight of AMOP was 192.5 kg/mol, its DE was 12.76%, and the DM was 1.92%, indicating that it is a naturally low-ester pectin. The DE of AMOP, determined via infrared spectroscopy, was 13.79%, further confirming that the extracted AMOP is a low-ester pectin. SEM and AFM observations show the linear structure of the AMOP molecule. A study on the gel properties of AMOP has shown that the concentrations of pectin, sucrose, pH, and Ca^2+^ affected the gel performance of AMOP. Ca^2+^ was determined to be a key factor in the formation of the AMOP gel, whereas sucrose was not a key factor, but adding sucrose could significantly increase the gel hardness. Under the conditions of pH 4, a Ca^2+^ concentration of 30 mg/g, a pectin concentration of 2%, and a sucrose concentration of 40%, the AMOP gel hardness was strongest, with a value of 128.87 g.

Sweet potato residue is a byproduct of sweet potato starch processing, and it has a high pectin content, making it a potential alternative raw material for commercial pectin production. This study compared the effects of different extractants on the extraction of pectin from high-quality sweet potato samples. For the first time, it was clearly demonstrated that sweet potato pectin is of the RG-I type with low esterification. The gel properties of sweet potato pectin were investigated to provide fundamental data for the commercial production and application of sweet potato pectin.

## Figures and Tables

**Table 1:** Extraction rate and basic components of sweet potato pectin extracted using different extractants.

Extractant	Dosage	Extraction Rate (%)	Ash Content (%)	Protein (%)	Mw (kg/mol) *
Trisodium citrate	0.50%	10.34 ± 0.22 ^h^	5.25 ± 0.01 ^c^	1.50 ± 0.05 ^cd^	174.7 ± 2.0 ^c^
	0.80%	12.96 ± 0.17 ^c^	5.37 ± 0.08 ^b^	1.55 ± 0.03 ^abc^	
	1.00%	12.05 ± 0.07 ^f^	5.52 ± 0.08 ^a^	1.58 ± 0.015 ^a^	
Ammonium oxalate	0.50%	12.80 ± 0.11 ^d^	2.16 ± 0.04 ^i^	1.45 ± 0.03 ^f^	182.5 ± 2.2 ^a^
	0.80%	14.42 ± 0.08 ^a^	2.30 ± 0.05 ^h^	1.50 ± 0.02 ^cd^	
	1.00%	13.71 ± 0.10 ^b^	2.45 ± 0.06 ^g^	1.56 ± 0.02 ^ab^	
Disodium hydrogen phosphate	0.50%	9.85 ± 0.07 ^i^	4.47 ± 0.05 ^f^	1.46 ± 0.04 ^de^	176.0 ± 2.1 ^b^
	0.80%	12.42 ± 0.03 ^e^	4.62 ± 0.02 ^e^	1.52 ± 0.03 ^bc^	
	1.00%	11.61 ± 0.05 ^g^	4.82 ± 0.07 ^d^	1.55 ± 0.03 ^abc^	
Citric acid	pH 2	7.61 ± 0.05 ^l^	1.52 ± 0.02 ^j^	1.42 ± 0.02 ^f^	152.5 ± 1.9 ^d^
Hydrochloric acid	pH 2	8.91 ± 0.04 ^k^	1.49 ± 0.02 ^j^	1.46 ± 0.04 ^de^	144.9 ± 1.7 ^e^

^a–l^ Values followed by different letters in the same column are significantly different (*p* < 0.05). * Mw is the average molecular weight.

**Table 2:** Basic composition of ammonium oxalate-extracted purified sweet potato pectin (AMOP).

	AMOP
Moisture (%)	7.82 ± 0.15
Ash (%)	0.78 ± 0.21
Protein (%)	0.35 ± 0.10
Mw (kg/mol) *	192.5 ± 2.3

* Mw is the average molecular weight.

**Table 3:** Monosaccharide composition of ammonium oxalate-extracted purified sweet potato pectin (AMOP).

Monosaccharide Composition (mol%)	
Rha	6.12 ± 0.05 ^d^
Fuc	0.26 ± 0.02 ^f^
Ara	21.08 ± 0.20 ^c^
Gal	29.84 ± 0.10 ^b^
Glc	1.69 ± 0.07 ^e^
Gal-A	41.00 ± 0.10 ^a^
**Sugar molar ratios (mol%)**	
HG	34.88
RG-I	63.16
Rha/Gal-A	0.1
(Gal + Ara)/Rha	8.32

^a–f^ Values followed by different letters in the same column are significantly different (*p* < 0.05). Abbreviations: Rha, rhamnose; Fuc, fucose; Ara, arabinose; Gal, galactose; Glc, glucose; Gal-A, galacturonic acid; HG, homogalacturonan; and RG-I, rhamnogalacturonan-I

**Table 4:** Molecular weight, degree of esterification (DE), and methoxy content (DM) of ammonium oxalate-extracted purified sweet potato pectin (AMOP).

	AMOP
Mw (kg/mol) *	192.5 ± 2.3
Mn (kg/mol) *	174.7 ± 1.9
PD *	1.10
DE (%)	12.76 ± 1.02
DM (%)	1.92 ± 1.02

* Mw is the molecular weight average, * Mn is the average molecular weight, and * PD is the polydispersity index.

**Table 5:** Effect of pectin concentration on the texture of ammonium oxalate-extracted purified sweet potato pectin (AMOP) gels.

Concentration (%)	Hardness (g)	Stickiness (mJ)	Elasticity (mm)	Cohesiveness	Chewiness (mJ)
0.5	4.60 ± 0.72 ^d^	0.01 ± 0.00 ^d^	1.80 ± 0.35 ^b^	0.31 ± 0.90 ^b^	1.82 ± 0.05 ^e^
1	41.30 ± 0.85 ^c^	0.09 ± 0.12 ^c^	3.65 ± 0.13 ^a^	0.48 ± 0.10 ^a^	5.21 ± 0.06 ^d^
1.5	80.43 ± 9.70 ^b^	0.14 ± 0.38 ^b^	3.82 ± 0.96 ^a^	0.48 ± 0.21 ^a^	7.76 ± 017 ^b^
2	102.20 ± 7.74 ^a^	0.20 ± 0.01 ^a^	3.93 ± 0.55 ^a^	0.52 ± 0.05 ^a^	8.16 ± 0.14 ^a^
2.5	99.53 ± 3.76 ^a^	0.15 ± 0.15 ^b^	3.72 ± 0.68 ^a^	0.49 ± 0.36 ^a^	7.36 ± 0.22 ^c^

^a–e^ Values followed by different letters in the same column are significantly different (*p* < 0.05).

**Table 6:** Effect of Ca^2+^ concentration on the texture of ammonium oxalate-extracted purified sweet potato pectin (AMOP) gels.

Ca^2+^ (mg/g)	Hardness (g)	Stickiness (mJ)	Elasticity (mm)	Cohesiveness	Chewiness (mJ)
10	29.67 ± 3.11 ^c^	0.09 ± 0.01 ^d^	3.23 ± 0.12 ^c^	0.48 ± 0.02 ^c^	6.35 ± 0.05 ^e^
20	56.80 ± 2.84 ^b^	0.14 ± 0.01 ^c^	3.87 ± 0.12 ^ab^	0.55 ± 0.02 ^b^	6.52 ± 0.03 ^d^
30	105.39 ± 2.95 ^a^	0.3 ± 0.03 ^a^	4.31 ± 0.18 ^a^	0.65 ± 0.06 ^a^	8.90 ± 0.08 ^a^
40	102.20 ± 7.74 ^a^	0.20 ± 0.01 ^b^	3.93 ± 0.55 ^ab^	0.52 ± 0.05 ^bc^	8.72 ± 0.14 ^c^
50	103.37 ± 1.72 ^a^	0.09 ± 0.08 ^d^	3.76 ± 0.42 ^b^	0.48 ± 0.02 ^c^	8.66 ± 0.06 ^b^

^a–e^ Values followed by different letters in the same column are significantly different (*p* < 0.05).

**Table 7:** Effect of pH on the texture of ammonium oxalate-extracted purified sweet potato pectin (AMOP) gels.

pH	Hardness (g)	Stickiness (mJ)	Elasticity (mm)	Cohesiveness	Chewiness (mJ)
2	25.20 ± 0.40 ^e^	0.23 ± 0.12 ^c^	4.22 ± 0.02 ^a^	0.45 ± 0.04 ^b^	1.67 ± 0.07 ^e^
3	39.20 ± 0.30 ^d^	0.24 ± 0.01 ^c^	4.26 ± 0.05 ^a^	0.62 ± 0.02 ^a^	5.52 ± 0.08 ^d^
3.5	102.69 ± 0.41 ^b^	0.27 ± 0.15 ^ab^	4.28 ± 0.01 ^a^	0.63 ± 0.01 ^a^	6.53 ± 0.49 ^c^
4	105.39 ± 2.95 ^a^	0.30 ± 0.03 ^a^	4.31 ± 0.18 ^a^	0.65 ± 0.06 ^a^	8.89 ± 0.08 ^a^
5	102.58 ± 2.26 ^b^	0.26 ± 0.06 ^bc^	4.29 ± 0.02 ^a^	0.46 ± 0.09 ^b^	8.84 ± 0.06 ^a^
6	88.20 ± 0.32 ^c^	0.25 ± 0.06 ^bc^	4.26 ± 0.06 ^a^	0.42 ± 0.02 ^b^	8.56 ± 0.04 ^a^
7	85.71 ± 0.94 ^c^	0.23 ± 0.06 ^c^	4.24 ± 0.01 ^a^	0.42 ± 0.01 ^b^	8.15 ± 0.13 ^b^

^a–e^ Values followed by different letters in the same column are significantly different (*p* < 0.05).

**Table 8:** Effect of sucrose concentration on the texture of ammonium oxalate-extracted purified sweet potato pectin (AMOP) gels.

Sucrose (%)	Hardness (g)	Stickiness (mJ)	Elasticity (mm)	Cohesiveness	Chewiness (mJ)
0	105.39 ± 2.95 ^e^	0.30 ± 0.03 ^bc^	4.31 ± 0.18 ^a^	0.65 ± 0.06 ^a^	8.90 ± 0.08 ^a^
10	110.27 ± 0.30 ^d^	0.29 ± 0.02 ^c^	4.32 ± 0.07 ^c^	0.56 ± 0.03 ^b^	8.92 ± 0.08 ^d^
20	114.80 ± 0.12 ^c^	0.31 ± 0.03 ^bc^	4.46 ± 0.04 ^bc^	0.51 ± 0.03 ^b^	9.27 ± 0.06 ^c^
30	120.60 ± 0.79 ^b^	0.33 ± 0.03 ^bc^	4.00 ± 0.06 ^d^	0.50 ± 0.03 ^b^	10.32 ± 0.03 ^b^
40	128.87 ± 0.48 ^a^	0.63 ± 0.03 ^a^	4.62 ± 0.13 ^a^	0.66 ± 0.05 ^a^	11.72 ± 0.09 ^a^
50	120.00 ± 0.95 ^b^	0.35 ± 0.04 ^b^	4.52 ± 0.07 ^ab^	0.52 ± 0.02 ^b^	10.41 ± 0.06 ^b^

^a–e^ Values followed by different letters in the same column are significantly different (*p* < 0.05).

## Data Availability

Data are contained within the article.
